# Electrophysiological correlates of masked orthographic and phonological priming in Chinese–English bilinguals

**DOI:** 10.1038/s41598-022-21072-z

**Published:** 2022-10-05

**Authors:** Er-Hu Zhang, Jiaxin Li, Xin-Dong Zhang, Defeng Li, Hong-Wen Cao

**Affiliations:** 1 Research Center for Language, Cognition and Language Application, Chongqing University, Campus D of Chongqing University, No. 55 Huxi Southern Road, Shapingba District, Chongqing, 401331 People’s Republic of China; 2 Centre for Studies of Translation, Interpreting and Cognition, University of Macau, Taipa, Macau SAR People’s Republic of China

**Keywords:** Cognitive neuroscience, Language

## Abstract

Extensive behavioral and electrophysiological evidence has demonstrated that native translations are automatically activated when bilinguals read non-native words. The present study investigated the impact of cross-language orthography and phonology on Chinese–English bilingual lexicons with a masked priming paradigm. The masked primes and targets were either translation equivalents (TE), orthographically related through translation (OR), phonologically related through translation (PR), or unrelated control (UC). Participants retained the targets in memory and decided whether the delayed catch words matched the targets. ERP data showed significant masked translation priming effects, as reflected by decreased ERP amplitudes in the TE condition in the 300–600 ms time window from frontal to parietal electrode clusters. Importantly, compared with the UC condition, the PR rather than OR condition elicited less negative ERP waveforms in the 300–500 ms time window with a frontal distribution. Taken together, these temporal and spatial dynamics suggested an automatic cross-language co-activation at the phonological and semantic levels for different-script bilinguals.

## Introduction

The question of how bilinguals process and access lexical-semantic representations has been of considerable interest in bilingualism. Bilingual connectionist models, such as the Bilingual Interactive Activation model (BIA model)^1^, Bilingual Interactive Activation plus model (BIA+model)^2^, and multilink model^3^, proposed that the orthographic, phonological and semantic representations of 2 languages in bilinguals’ lexicon were integrated and interconnected. Indeed, ample evidence in studies with cognate words, interlingual homophones, or interlingual homographs^4–7^ suggested that the lexical representations from one language were interactively co-activated in a bottom-up manner when bilinguals read in another language, leading to the non-selective lexical access. This evidence is based on the fact that a briefly presented masked prime in one language usually facilitates a target word recognition in the other language.

Furthermore, these findings have been also generalized to different-script bilinguals, such as Korean–English bilinguals^8^, Japanese–English bilinguals^9–11^, Chinese–English bilinguals^12^, and Russian–English bilinguals^13,14^. In the investigations of cross-language priming with different categories of bilinguals, previous studies have revealed the priming effects and automatic interplay between L1 and L2 orthographic, phonological and semantic representations^8–17^. For example, the response latencies of lexical decision to English targets were facilitated by phonologically similar Japanese primes. Researchers argued that the phonological representations of English and Japanese were integrated despite the orthographic differences between them^9^.

Similar findings showing L1–L2 co-activation at phonological level have been found in neurophysiological studies. For example, by manipulating the critical prime (L1, Russian)-target (L2, English) word pairs for phonological (S−P+), semantic (S+P−), or both phonological and semantic similarity (S+P+), Novitskiy and colleagues^13,14^ found the semantic and phonological interplay between L1 and L2, as reflected by the modulation of N400 component, a well-established component that has been shown to index lexical-semantic integration in the 300–500 time window^18,19^. The N400 amplitude typically reflects the ease or difficulty of integrating the orthographic, phonological or semantic knowledge relative to a word^20,21^. Thus, any factor that boosts lexical access should reduce the N400 amplitude. Concretely, relative to unrelated control (S−P−), S−P+ elicited reduced negativity in the N400 component, whereas no difference was observed between S+P+ and S + P−. This interplay indicates the highly interactive nature of the language system.

Moreover, masked priming studies have also tried to disentangle the roles of cross-language orthography and phonology in bilingual word processing^15–17^. For example, in a lexical decision to L1/L2 targets with masked L2/L1 primes in Greek (L1)–Spanish (L2) bilinguals, critical prime-target word pairs were either orthographically and phonologically related (O+P+) or phonologically related (O−P+). Relative to the unrelated control condition, behavioral results revealed bidirectional masked priming for O–P+ condition but not for O+P+ condition. Those results suggested that the automatic co-activation of cross-language phonological codes was strongly influenced by the orthographic properties of the input^15^. In other two similar word naming tasks with masked primes^16,17^, even though cross-language phonological but not orthographic overlap facilitated naming reaction times, electrophysiological data provided evidence that the cross-language effect of masked L2 on L1 word naming occurred at both orthographic and phonological levels. For example, researchers investigated the cross-language masked onset priming effect with prime (L2, English)-target (L1, Russian) word pairs that were orthographically, phonologically or both orthographically and phonologically similar. The impact of L2 orthography and phonology on L1 word naming was observed in the 150–250 ms and 250–450 ms time windows, respectively, as reflected by less negative waveforms^16^. The evidence, taken together, indicated that both types of lexical code (i.e., orthography and phonology) could contribute to the bilingual lexical interaction in a bottom-up manner, attesting to the bilingual interactive-activation models.

It is worth noting that a potential controversy in the above studies is that any effects of the masked primes on target processing may simply be the result of solely visual or acoustic feature overlap^22^. That is, those masked priming effects may be caused by cross-language co-activation, physical overlap, or both cross-language activation and physical overlap. However, such studies do not effectively distinguish these effects. More compelling evidence comes from studies investigating cross-language lexical effects through translation, especially for different-script Chinese–English bilinguals. Numerous behavioral and electrophysiological studies have demonstrated the automatic and unconscious activation of Chinese translations during the processing of English words, even in a purely English word context. For example, in a behavioral masked priming task conducted by Zhang et al.^23^, Chinese–English bilinguals performed a lexical decision to English target words, preceded by masked English primes. The participants did not realize that the Chinese two-character translations of the critical English word pairs hid first- or second-morpheme overlap. Relative to unrelated control, behavioral results revealed faster response time for critical word pairs whose Chinese translations contained the first-morpheme repetition (e.g., east[**东**/**Dong1**/]-thing[**东**西/**Dong1**Xi1/]). In a series of masked priming studies by Wen and van Heuven^24^, even though researchers did not replicate the result by Zhang, van Heuven, and Conklin^23^ (Exp 2), the masked prime effect occurred for English target words paired with masked Chinese words that shared partial repetition with the translations of the targets (e.g., “**事**业/**Shi4**Ye/”[career]-fact[**事**实/**Shi4**Shi2/]) (Exp 3). Additionally, Thierry and Wu^25^ asked Chinese–English bilinguals and English monolinguals to decide whether visible English words presented in pairs were related in meaning or not; they were unaware of the relationship that some of the semantically unrelated word pairs contained a character repetition (i.e., orthographic and phonological repetition) in their Chinese translations (e.g., train[**火**车/**Huo3**Che1/]-ham[**火**腿/**Huo3**Tui3/]). Even though behavioral results did not reveal any priming effects, the hidden factor significantly modulated the N400 component in Chinese–English bilinguals, but not in English monolinguals, as reflected by less negative N400 amplitudes in hidden factor condition than in unrelated control condition. Accordingly, researchers explained that bilinguals automatically and unintentionally translated English words into Chinese and then the lexical-semantic overlap between translations facilitated the processing, resulting in reduced N400 amplitude. Taken together, these findings provided clear evidence of the automatic activation of Chinese translations in the processing of English words.

Furthermore, following the investigation by Thierry and Wu^25^, Wu and Thierry^26^ further directly differentiated whether orthographic and/or phonological representations of Chinese words were unconsciously activated during recognition of English words. In the same implicit priming paradigm, researchers manipulated the critical English word pairs which contained either an orthographic (e.g., accountant[**会**计/**Kuai4**Ji4]-conference[**会**议/**Hui4**Yi4/]) or a phonological (e.g., experience[**经**验/**Jing1**Yan4/]-surprise[**惊**讶/**Jing1**Ya4/]) repetition in the Chinese translations. The pattern of ERP results (reduced N400 amplitude) demonstrated that processing English words automatically activated the phonological but not the orthographic representations of Chinese translations. In contrast, Ma and Ai^27^ asked Chinese–English bilinguals to judge whether the L2–L1 word pairs were translation equivalents or not. The focus was on the judgment performance of participants rejecting the translation non-equivalents that were related in orthography (e.g., cup[杯/Bei1/]-“坏/Huai4/”[bad]) or phonology (cup[杯/Bei1/]-“悲/Bei1/”[sad]) through translation. The results showed that bilinguals with lower and higher proficiency were poorer in speed and accuracy in rejecting orthographic rather than phonological distractors. Researchers thus claimed that the orthographic but not phonological representation of the Chinese translation words was activated. It is essential to point out that these results in visible recognition tasks may be led by strategic translation rather than unconscious lexical interactivity. Issues related to electrophysiological correlates of masked cross-language orthographic and phonological priming remain unsolved.

Overall, following the logic of the masked priming mentioned above, the present study investigated the distinct roles of cross-language orthography and phonology in a masked priming task with Chinese–English bilinguals. Referring to the manipulations by Ma and Ai^27^, we choose monosyllabic Chinese words as masked primes, which have a clear dissociation between orthography and phonology, and English words as targets. Accordingly, the natural relationships between masked primes and targets could be translation equivalents (TE), orthographically related through translation (OR), phonologically related through translation (PR), or unrelated control (UC). Additionally, we employed a matching-to-sample judgment task^13,14,28^, in which a delayed catch word was represented after prime-target onset. Participants were instructed to retain the targets in memory and decide whether the delayed catch words matched the targets. Accordingly, this variant of the masked priming task could minimize motor-related artifacts on ERP data.

We predicted similar behavioral outcomes to matching-to-sample judgment studies^13,14^ in which match condition exhibited faster response times and higher accuracy than mismatch condition. Additionally, we also predicted two alternative outcomes for the impact of prime-target relationships on delayed match judgment performance. If the subliminal prime-target relationships influenced the delayed match judgment performance, we would also observe an interaction between prime-target condition and target-match condition; if not, the interaction would not be observed.

Thus far, previous ERP studies investigating cross-language lexical-semantic priming with different categories of word pairs (e.g., translation equivalents, interlingual homophones, or interlingual homographs) have demonstrated the automatic L1–L2 co-activation when reading in one language, as reflected by reduced ERP waveforms in the critical conditions than in the unrelated control condition^10,13,14,16,17,29,30^. These effects have been found in both early (about 100–300 ms) and late (about 300–500 ms) time windows, therefore reflecting cross-language interplay at early as well as late stages of word processing. Based on these results, we expected to see similar reduced negativities in the ERP waveforms for the three through-translation priming conditions (i.e., TE, OR, and PR conditions) compared to UC condition in early and late time windows. We also predicted that the masked cross-language lexical effects would differ across different topographic regions according to previous bilingual literature^16,17^. Additionally, if indeed only one type of lexical code of Chinese is activated in the processing of English words as suggested by Ma and Ai^27^ or Wu and Thierry^26^, a priming effect in ERP response would be expected for English targets paired with Chinese primes which share orthography or phonology with the correct translations of targets, as reflected by less negative ERP response. Alternatively, if both Chinese orthography and phonology are activated in the processing of English words as suggested by the aforementioned bilingual interactive-activation models, we would also potentially observe the more widespread priming effects in ERP response in both orthographically and phonologically related word pairs.

## Results

### Behavioral results

Accuracy (ACC) and reaction times (RTs) were measured from the Target-Catch judgment. Two-way ANOVA with the factors of Prime-Target condition and Target-Catch condition revealed that the overall ACC of the match condition was higher than that of the mismatch condition, 94.94% ± 3.23% versus 93.05% ± 4.53%, *F*(1,28) = 6.47, *p* < 0.05, $\upeta_{{\text{p}}}^{2}$ = 0.19. No significant main effect of Prime-Target condition and interaction effect were observed, both *Fs* < 1.78, *p* > 0.05.

**Figure 1:**
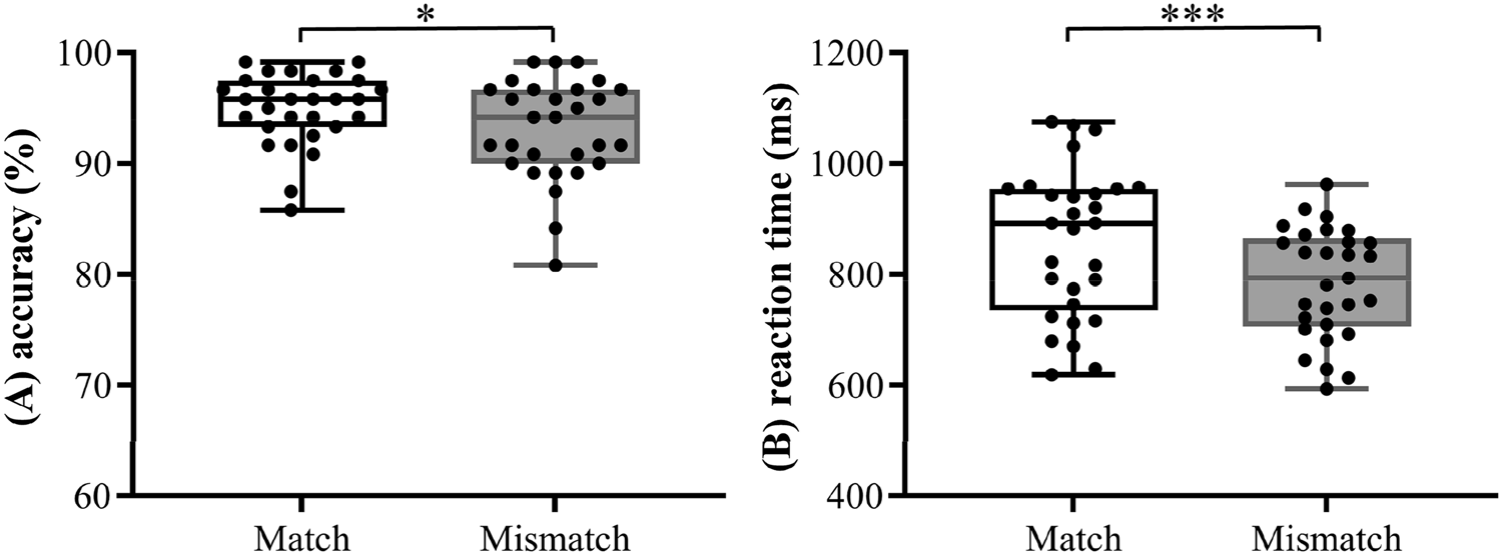
The boxplots of overall accuracy (**A**) and reaction times (**B**) in the Target-Catch match and mismatch conditions. The asterisk marks statistically significant differences (*p < 0.05, ***p < 0.001).

For average RTs, trials with errors (6.01% of the data) and RTs that were longer than 2000 ms or that were 2.5 standard deviations (SD) above or below mean RT (1.94% of the data) were discarded to reduce the outliers. The ANOVA revealed that the overall RT of the match condition was slower than that of the mismatch condition, 858 ms ± 134 ms versus 785 ms ± 99.5 ms, *F*(1,28) = 43.39, *p* < 0.001, $\upeta_{{\text{p}}}^{2}$ = 0.61. Also, no significant main effect of Prime-Target condition and interaction effect were observed, both *Fs* < 2.47, *p* > 0.05. Figure 1 showed the overall ACC and RTs for match and mismatch conditions due to the fact that the ANOVA did not reveal any significant main effects of Prime-Target condition and interaction effects between Prime-Target condition and Target-Catch condition on ACC and RT.

### ERP results

In the time windows of 0–100 ms, 100–200 ms, and 200–300 ms, the ANOVA did not reveal any significant main effect of Prime-Target condition, all *Fs* < 1.20, *p* > 0.05, nor interaction effects between Prime-Target condition and electrode cluster, all *Fs* < 1.28, *p* > 0.05, indicating no significant differences among the four Prime-Target conditions. Significant results are described in detail in the following paragraphs.

#### 300–400 ms

**Figure 2:**
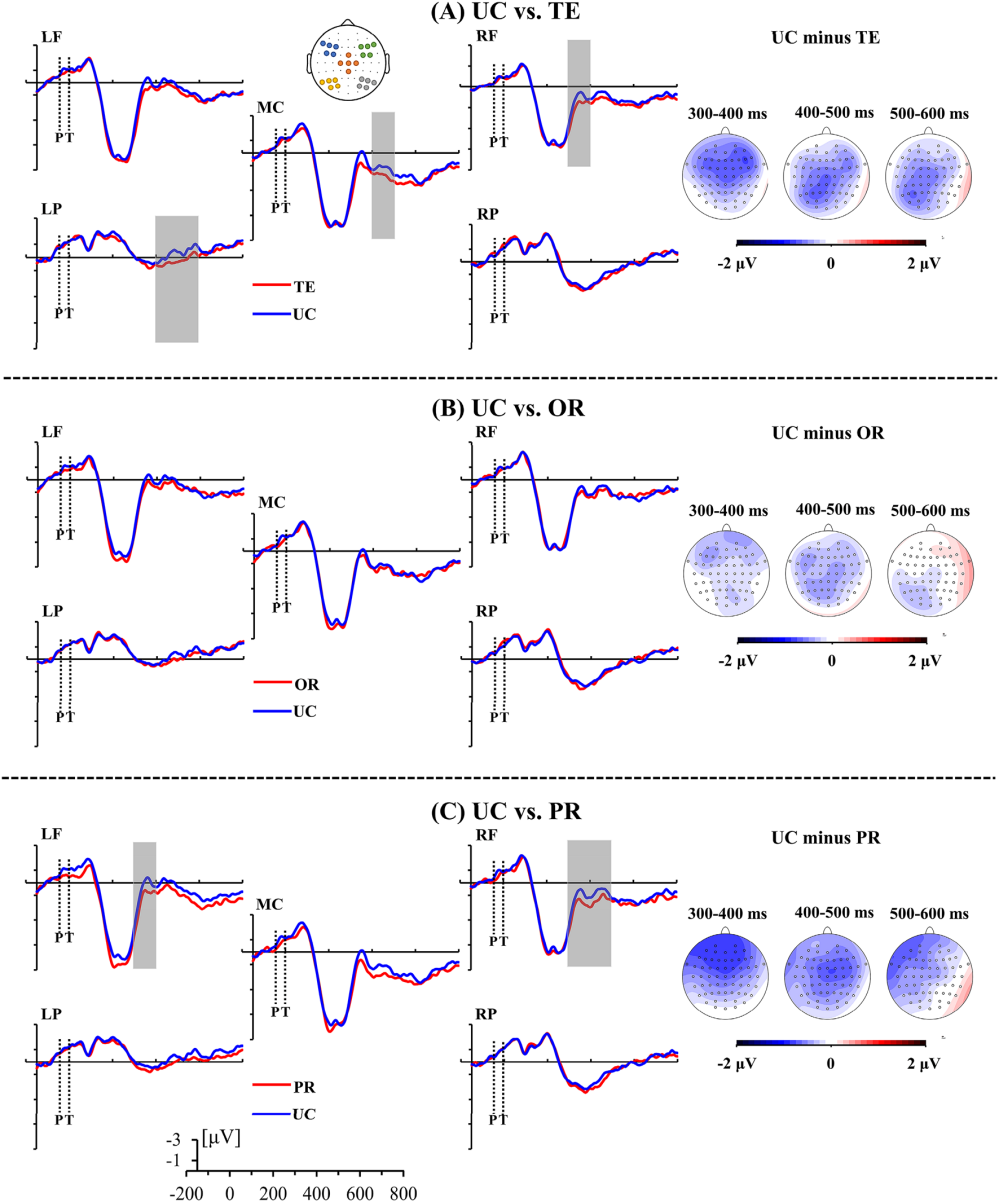
Grand averaged ERP waveforms elicited by unrelated control (UC, blue line) condition paired with (**A**) translation equivalent (TE, red line) condition, (**B**) orthographically related through translation (OR, red line) condition, and (**C**) phonologically related through translation condition (PR, red line). The gray rectangles indicate significant ERP differences across time windows and scalp regions analyzed (*p* < 0.05). The scalp voltage maps on the right correspond to the difference waves of unrelated control minus translation-related priming conditions in the 300–600 ms. *Note* P = prime, T = target; LF = left frontal, RF = right frontal, MC = middle central, LP = left parietal, RP = right parietal.

The ANOVA showed a significant main effect of Prime-Target condition, *F*(3,84) = 4.68, *p* < 0.01, $\upeta_{{\text{p}}}^{2}$ = 0.14. Further planned comparisons were made collapsing the electrode cluster factors. The results showed that both TE, *t*(28) = 2.80, *p* = 0.05, and PR, *t*(28) = 3.36, *p* < 0.05, conditions elicited less negative or marginally less negative ERP waveforms than the UC condition (see Fig. 2A,C, respectively). No difference was observed between OR and UC conditions, *t*(28) = 1.53, *p* > 0.05 (see Fig. 2B). Even though the two-way interaction effect between Prime-Target condition and electrode cluster was not significant, *F*(12,336) = 1.43, *p* > 0.05, $\upeta_{{\text{p}}}^{2}$ = 0.05, we predicted that the masked cross-language effects would differ across different electrode clusters according to previous bilingual literature^16,17^. Therefore, we performed planned comparisons between the three through-translation priming conditions and unrelated control in the five electrode electrodes, respectively. The results revealed that the differences between TE and UC conditions (see Fig. 2A), and between PR and UC conditions (see Fig. 2C) were mainly located in the frontal electrode clusters, all *ts* > 3.05, *p* < 0.05. No differences were observed in the central and parietal electrode clusters, all *ts* < 2.67, *p* > 0.05.

#### 400–500 ms

A significant main effect of Prime-Target condition was observed, *F*(3,84) = 4.46, *p* < 0.01, $\upeta_{{\text{p}}}^{2}$ = 0.14. Further planned comparisons collapsing the electrode cluster factor showed that both TE, *t*(28) = 3.21, *p* < 0.05, and PR, *t*(28) = 2.92, *p* < 0.05, conditions elicited less negative ERP waveforms than the UC condition (see Fig. 2A,C, respectively). No difference was found between OR and UC conditions, *t*(28) = 2.22, *p* > 0.05 (see Fig. 2B). In addition, the effects of Prime-Target condition were further explored across topographic regions despite the fact that the two-way interaction effect was not significant, *F*(12,336) = 0.71, *p* > 0.05, $\upeta_{{\text{p}}}^{2}$ = 0.03, pairwise comparisons analysis revealed that the difference between TE and UC conditions was mainly located in the left parietal, *t*(28) = 3.06, *p* < 0.05, and middle central, *t*(28) = 2.85, *p* < 0.05, electrode clusters (see Fig. 2A), and the difference between PR and UC conditions was mainly located in the right frontal electrode cluster (see Fig. 2C), *t*(28) = 3.10, *p* < 0.05. There were no significant differences in the rest of electrode clusters, all *ts* < 2.83, *p* > 0.05.

#### 500–600 ms

The main effect of Prime-Target condition, *F*(3,84) = 2.72, *p* = 0.049, $\upeta_{{\text{p}}}^{2}$ = 0.09, was significant. Further planned pairwise comparisons collapsing the electrode cluster factor showed that the TE condition elicited marginally less negative ERP waveforms than the UC condition, *t*(28) = 2.68, *p* = 0.07 (see Fig. 2A), and no differences between OR and UC conditions, t(28) < 1, *p* > 0.99, and between PR and UC conditions, t(28) = 1.76, *p* > 0.05, were observed (see Fig. 2B,C, respectively). Despite the fact that the two-way interaction effect was not significant, *F*(12,336) = 1.71, *p* > 0.05, $\upeta_{{\text{p}}}^{2}$ = 0.06, pairwise comparisons were performed to explore the effects of Prime-Target condition in the five electrodes clusters. The results revealed that the difference between TE and UC mainly came from the left parietal electrodes, t(28) = 4.00, *p* < 0.01 (see Fig. 2A). No differences were observed in the rest of electrode clusters, all *ts* < 2.62, *p* > 0.05.

## Discussion

The present study investigated the distinct roles of cross-language orthography and phonology in the masked priming paradigm with Chinese–English bilinguals using sensitive ERP measures. Regarding the delayed matching-to-sample judgment performance, the results differed from those of previous literature^13,14^ but revealed a typical speed-accuracy trade-off in which participants were more accurate but slower in response to the target-catch match condition than to the mismatch condition. Additionally, the Prime-Target relationships did not impact the later match judgment performance.

Compared with UC condition, ERP data showed significantly decreased ERP amplitudes in the TE condition, starting at 300 ms and continuing up to 600 ms after target onset from frontal to parietal electrode clusters (see Fig. 2A). Because there was no overt overlap between word pairs, the temporal and spatial dynamics indicated implicit lexical-semantic priming through translation. Specifically, English target words could be rapidly activated and boosted by the corresponding Chinese translation. These data are consistent with previous literature investigating the asymmetric pattern of masked translation priming effects^29,30^. For example, when Japanese–English bilinguals performed a semantic categorization task to target words paired with masked primes, Hoshino et al.^29^ found significant priming effects in the L1–L2 direction, as reflected by decreased N250 (200–350 ms) and N400 (350–550 ms) amplitudes, which have been associated with the grapheme-to-phoneme conversion and lexical-semantic processing in visual word processing, respectively^31^. Notably, Hoshino et al.^29^ also found a priming effect in the N/P150 (100–250 ms) component, which is thought to reflect the mapping of visual features onto prelexical orthographic representations^31^. The dissociation in time courses between our findings (300–600 ms) and Hoshino’s^29^ reports of earlier masked L1–L2 translation priming in the N/P150 and N250 time windows may result from the different experiment tasks performed. Unlike the current matching-to-sample judgment task, in which participants were asked to retain the targets in memory and decide whether the delayed catch words matched the targets, semantic categorization typically requires bilinguals to process the lexical-semantic representations of target words more deeply. Still, the robust L1–L2 translation priming effect suggested that the subliminally presented primes do facilitate the lexical-semantic processing of target words through translation, even if there is a change in script across primes and targets.

Moreover, the absence of OR effect (see Fig. 2B) and the appearance of PR effect in the 300–500 ms time window (see Fig. 2C) demonstrated that Chinese phonology but not orthography was automatically activated in English word processing, in line with the previous findings of phonology-based cross-language activation^26,32^. The 300–500 ms time window typically corresponds to the N400 component, which is a well-established neural correlate of lexical-semantic priming and integration in both monolingual and bilingual word processing^18,19,33^. For example, a reduced negativity in N400 amplitude was observed for phonologically similar but semantically unrelated L1–L2 word pairs in Russian–English bilinguals. Researchers interpreted it as a facilitation effect due to the fact that the masked primes pre-activated the phonological representations of targets^13^. Accordingly, the explanation for the PR effect might be that the English target words were activated by the phonological representations of Chinese primes. Besides, compared with the studies by Wu and Thierry^26^ in which English word pairs shared character-level orthographic representation (e.g., accountant[**会**计/**Kuai4**Ji4/]-conference[**会**议/**Hui4**Yi4/]), the Chinese–English pairs in the present study shared sub-character orthography (e.g., “鲜/Xian1/”[fresh]-whale [鲸/Jing1/], where the Chinese words “鲜” and “鲸” share the same radical “鱼”). Thus, the more general conclusion is that the cross-language orthographic activation did not occur at the sub-character and character level.

According to the topographical distribution of phonological priming effects in the two time windows (see Fig. 2C), the cross-language phonological processing might be driven by neural structures located in frontal areas. This pattern is in line with the neuroimaging studies investigating phonological processing during visual word recognition in which that activation of the inferior frontal cortex (IFC) is involved in lexical-phonological processing^34^. Additionally, it is also important to point out that the monolingual masked priming paradigm concerning the time course of orthographic and phonological code activation of visual word recognition has revealed that the orthographic and phonological effects were located in the parietal and frontal sites, respectively^35^. Furthermore, in the investigation of the influence of English (L2) orthography and phonology on Russian (L1) word naming, the cross-language orthographic and phonological effects were also mainly located in the parietal and frontal sites, respectively^16^. Dimitropoulou and colleagues^15^ demonstrated that the automatic co-activation of cross-language phonological codes significantly interacted with the orthographic properties of the input. These results fit with the theoretical framework of the Interactive Activation model (IA model)^36,37^ and its bilingual extensions (BIA model, BIA+ model, and multilink model)^1–3^. Because these models suggested that bidirectional and strong links between orthography and phonology allow for automatic activation of orthographic/phonological codes whenever phonology/orthography is accessed. However, it is necessary to reconsider to what extent the assumptions of these models can be generalized to different-script bilinguals due to the absence of OR effect in the present study. Baddeley and Hitch^38^ proposed the phonological loop including phonological store and articulatory rehearsal in the Working Memory Model, and assumed that short term memory retention was likely to rely more on phonological information than orthographic information. This possibly explains why phonology plays a stronger role than orthography. However, whether these assumptions can be generalized to bilingual processing needed to be further explored.

Note that two possible temporal routes, lexical-level bidirectional links or semantic feedback, might be responsible for the PR priming effect. Specifically, the L2 words might be co-activated by the corresponding L1 phonology through a direct lexical link. An alternative explanation might be that the L2 words could rapidly activate the semantics and subsequently feed activation back to the phonological representations of their translation equivalents through the meaning to the lexical route. Next, the L1 phonology facilitated the co-activation of L2 words. Guo et al.^39^ tried to explore the time course of accessing the meaning of English words with a translation recognition task. Chinese–English bilinguals were asked to decide whether Chinese words were the correct translations of preceding English words (L2–L1) at a 750 ms stimulus onset asynchrony (SOA = 750 ms, Exp 1), and one type of the critical translation non-equivalents was related in lexical form through translation (e.g., bee[蜂/Feng1/]-“峰/Feng1/”[peak]). Compared with the unrelated control condition, through-translation form distractors elicited a larger early P200 (150–300 ms) component and late positive component (LPC, 500–700 ms). The P200 and LPC are usually thought to reflect the perceptual orthographic and/or phonological overlap between word pairs^40,41^ and pre-response reanalysis or checking process^42,43^, respectively. However, at a 300 ms SOA (Exp 2), the early P200 priming effect disappeared, which suggested that the lexical acquisition (orthography and/or phonology) of L1 in the processing of L2 was relatively late, possibly through semantic feedback.

In addition, different bilingual models have also tried to explain the temporal dynamics of bilingual lexical access. The BIA+ model^2^ accounts for an integrated word recognition system with subsystems to deal with lexical interplay across languages. It suggested that cross-language lexical priming in bilinguals did arise via bidirectional lexical links and top-down conceptual feedback, attesting to the dynamic and highly interactive nature of the language system. Thus, both routes (connections between lexicons or semantic feedback) work in concert and drive the patterns between languages. Additionally, according to the influential Revised Hierarchical Model (RHM)^44^, the two potential routes or their relative influence depend on the L2 proficiency, with lower proficiency bilinguals relying more on the direct lexical links and higher-proficiency bilinguals more on the semantics feedback. Moreover, on the basis of connectionist models^1–3^, Wen and van Heuven^24^ proposed the Chinese–English Interactive Activation Model (CE-IAM), which supposes that languages with different scripts are connected and interact with each other only through the shared semantic layers. The current experiment design could not tease apart the temporal routes or their relative influence. Future studies need to further explore the temporal dynamics of cross-language interactions in the processing of different-script L1/L2 words.

There are a few limitations in the present study that can be improved or explored in future studies. First of all, we did not give the same task to monolingual English participants as previous studies^25,26^. A direct comparison of monolingual and bilingual participants will strengthen the interpretations of the present study. Furthermore, previous studies on same-script bilingual language processing (e.g., interlingual homographs) have demonstrated parallel orthographic co-activation of two languages. However, the absence of the orthographic effects in the present study cannot be explained in the framework of current bilingual connectionist models. Finally, despite the lack of significant interactions between Prime-Target condition and topographic factor, we mentioned a frontal distribution bias for the cross-language phonological priming effect. This pattern might due to the relatively lower spatial resolution of ERP measures. Recent Chinese neuroimaging studies demonstrated that phonological processing involved multiple brain areas^45–47^, such as left inferior parietal lobule and right superior temporal gyrus. Moreover, a meta-analysis with eight fMRI studies revealed a bilateral involvement of the ventral occipito-temporal regions for both phonological and semantic processing^46^. It is essential to further investigate the spatial dynamics of the masked cross-language lexical priming with better spatial resolution measures, such as fMRI or MEG. This will provide us a more comprehensive picture of the bilingual networks.

In conclusion, the present study provides direct evidence that through-translation masked priming effects do occur at the lexical-semantic level in different-script Chinese–English bilinguals. More specifically, a significant masked translation priming was observed, as reflected by reduced ERP waveforms starting at 300 ms and continuing up to 600 ms and elicited from frontal to parietal electrode clusters. More importantly, compared with unrelated control, through-translation related word pairs in phonology but not in orthography also elicited less negative ERP waveforms in the 300–500 ms at the frontal electrode cluster. Despite above-mentioned limitations, these temporal and spatial dynamics could serve as strong evidence of an automatic cross-language phonological and semantic activation for Chinese–English bilinguals, besides the dissociation found with orthography, a processing level that, in the context of present study, is revealed as not affected by such L1–L2 interactivity.

## Methods

### Participants

Thirty-five Chinese (L1)–English (l2) bilinguals (17–25 years, mean age = 20 ± 2.1 years old, 21 males) were paid to participate in the experiment. All bilinguals were right-handed and had normal or corrected-to-normal vision. Written informed consent was obtained from all subjects before experiment. The whole experimental protocol was examined and approved by the ethical committee of the Chongqing University, and performed in accordance with relevant guidelines and regulations. Three participants were excluded due to poor EEG signals.

All bilinguals began formal English language learning in the classroom at around the age of 10. The English proficiency of bilinguals was evaluated as follows: First, a self-assessment questionnaire of English listening, speaking, reading, and writing skills was administered to all bilinguals on a 5-point Likert scale, with 1 indicating not fluent and 5 indicating very fluent. The self-reporting mean scores for English listening, speaking, reading, and writing skills were 3.0 (*SD* = 0.6), 2.9 (*SD* = 0.5), 3.5 (*SD* = 0.7), and 3.1 (*SD* = 0.6), respectively. Second, they scored an average of 526 (*SD* = 47) in the TEM4 (Test for English Majors-Band 4, the passing score and highest score are 425 and 710, respectively). Finally, all bilinguals took the English version of the LexTALE test downloaded from www.lextale.com. The LexTALE test is an English vocabulary decision-making task consisting of 60 items, including 40 words and 20 nonwords. The task of the participant was to decide whether each item was an existing English word or not. Accordingly, LexTALE is an effective test of English vocabulary that correlates well with the measure of general English proficiency^48^. The mean LexTALE score was 54.8 (*SD* = 8.5, highest score is 100, below 59 indicates relatively lower proficiency). Taken together, these results revealed that participants were all intermediate unbalanced Chinese–English bilinguals. The summary of the participants' English language proficiency were shown in Table 1.

**Table 1:** Summary of the participants’ mean self-assessed English proficiency, CET 4 score, and LexTALE test score (M ± SD and Min–Max).

		Mean (SD)	Min–Max
Mean self-assessed English proficiency	Listening	3.0 (0.6)	2–4
	Speaking	2.9 (0.5)	2–4
	Reading	3.5 (0.7)	2–5
	Writing	3.1 (0.6)	2–4
TEM4 score		526 (47)	443–637
LexTALE test score		54.8 (8.5)	42.5–81.3

### Stimuli

We first generated a database of 240 English lowercase targets which were randomly subdivided into four lists of 60 items (see Online Appendix). Then, five postgraduate students who were Chinese–English bilinguals and did not participate in the formal experiment first translated the English words into Chinese, and then generated the corresponding Chinese primes into four Prime-Target conditions:

1. 60 word pairs of Chinese–English translation equivalents: “砖/Zhuan1/”-brick;
2. 60 word pairs of Chinese–English translation non-equivalents that were related in orthography through translation:“鲜/Xian1/”(fresh)-whale(鲸/Jing1/), where the Chinese words “鲜” and “鲸” share the same radical “鱼”;
3. 60 word pairs of Chinese–English translation non-equivalents that were related in phonology through translation: “瞎/Xia1/”(blind)-shrimp(虾/Xia1/), where the Chinese words “瞎” and “虾” share the same pronunciation/Xia1/;
4. 60 word pairs of Chinese–English translation non-equivalents that were orthographically, phonologically, and semantically unrelated through translation: “锅/Guo1/”(pot)-tooth(牙/Ya2/).

Finally, according to the English lowercase target words, a catch word list was generated that was formed of English uppercase words that match (e.g., brick-BRICK) or mismatch (e.g., train-TRAIL) with the English lowercase target words. Accordingly, the research design consists of two within-subject independent variables: Prime-Target condition (translation equivalent [TE] vs. orthographically related through translation [OR] vs. phonologically related through translation [PR] vs. unrelated control [UC]) and Target-Catch Match condition (match vs. mismatch).

According to the corpus of SUBTLEX-CH-WF^49^, all Chinese prime words were common one-character words with the number of strokes and the frequency of log10W (log10W is the log10 of the total count of words that has been observed in the corpus) calculated. One-way analyses of variance (ANOVA) revealed no significant differences in the number of strokes and frequency among the four Prime-Target conditions, *Fs* < 0.74, *p* > 0.05. Correspondingly, all English lowercase target words were highly used words with the string lengths ranging from 3 to 8 and with the frequency of log10W provided from the corpus of SUBTLEX_us_^50^. Also, no significant differences across the four conditions in string lengths and frequency were observed, *Fs* < 0.87, *p* > 0.05. The descriptive statistics (mean and *SD*) of the number of strokes, string lengths, and frequency across the four Prime-Target conditions are summarized in Table 2. Besides, the English uppercase catch words have the same string lengths as the target words, but the frequency of catch words in the Target-Catch match condition was significantly higher than that of catch words in the mismatch condition, 3.30 ± 0.49 versus 3.02 ± 0.93, paired-sample t-test, *t*(119) = 2.74, *p* < 0.01.

**Table 2:** The descriptive statistics (mean and *SD*) of the number of strokes, string lengths, and frequency (log10W) across the Prime-Target conditions.

L1–L2 relationships	Prime		Target	
	Number of strokes	Frequency	String length	Frequency
TE (“砖”—brick)	9.63 (4.02)	3.23 (0.41)	4.62 (1.03)	3.25 (0.55)
OR (“鲜”—whale[鲸])	9.15 (2.48)	3.22 (0.47)	4.43 (1.01)	3.31 (0.46)
PR (“瞎”—shrimp[虾])	9.32 (3.31)	3.32 (0.39)	4.55 (1.00)	3.32 (0.57)
UC (“锅”—tooth[牙])	8.82 (2.72)	3.27 (0.46)	4.70 (0.91)	3.39 (0.30)

### Procedure

**Figure 3:**
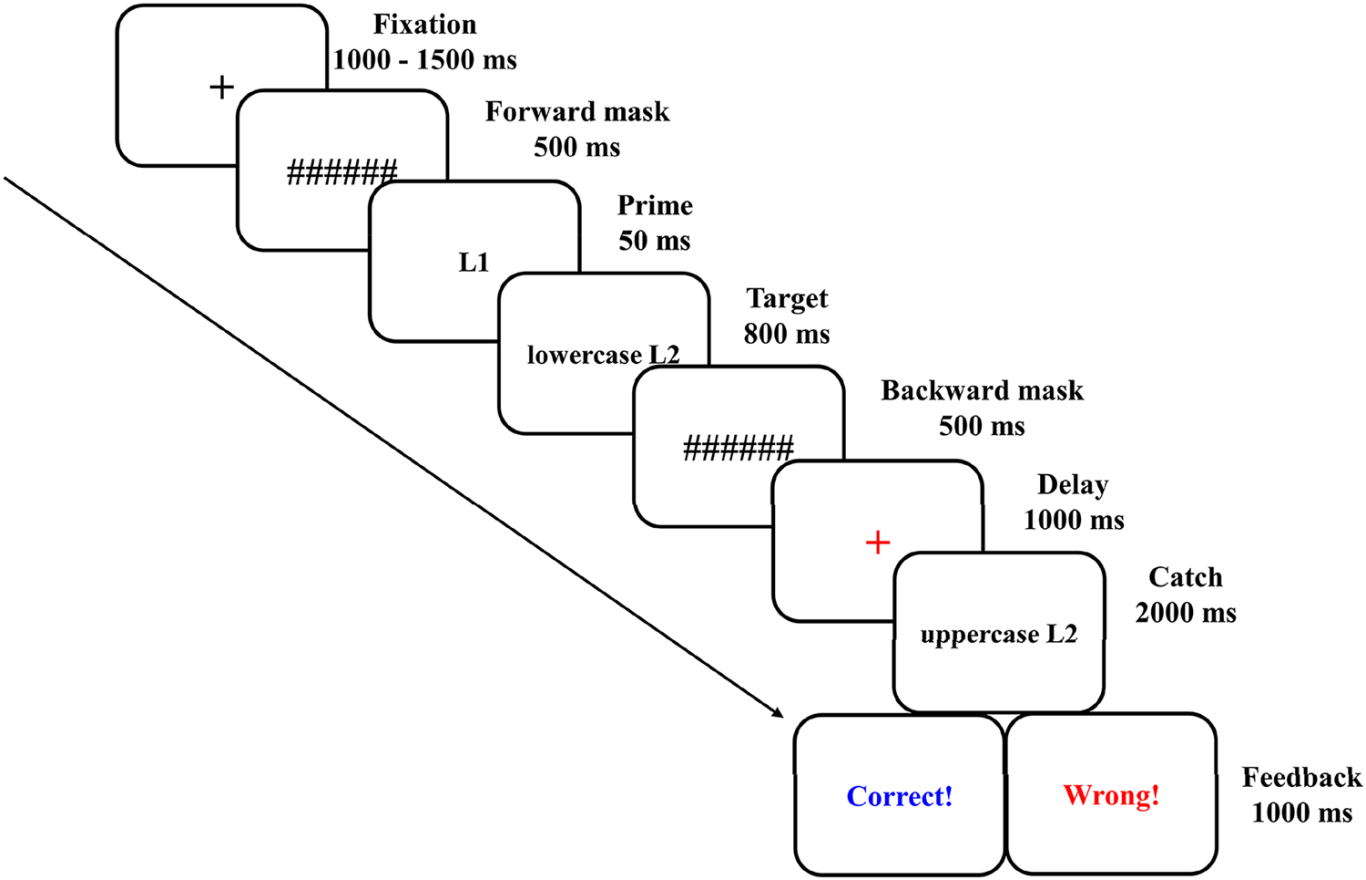
The schematic illustration of the masked prime paradigm used in the present experiment.

Participants were tested in an electrically shielded room, approximately 60 cm in front of a 19-inch monitor (refresh rate: 60 Hz). Participants were instructed to perform a matching-to-sample judgment task^13,14,28^, a variant of the masked priming task that could minimize motor-related artifacts on ERP data. Stimulus presentation was controlled by E-prime 3.0 software (Psychological Software Tools, Pittsburgh, PA.). Each trial (see Fig. 3) began with a black “+” fixation presented for a random duration of 1000–1500 ms. Then, a 500 ms forward mask (“######”, 50-point font), a 50-ms L1 prime (40-point font), an 800 ms lowercase L2 target (50-point font), and a 500-ms backward mask (“######”, 50 point font) were presented sequentially. Next, after a 1000-ms delay (a red “ + ” fixation), an L2 uppercase catch word was presented to a maximum of 2000 ms, and participants were instructed to remember the lowercase target words and judge whether the uppercase catch words were same as or different from the target words by pressing “F” or “J” (counterbalanced across participants). To ensure the participant’s attention on the L2 target words, 50% of the catch words were orthographically similar to the target words (mismatch, e.g., train-TRAIL), and another 50% of the catch words were the same as the target words (match, e.g., brick-BRICK). Finally, a 1000-ms feedback was presented on the screen. Accordingly, we obtained ERP data from Prime-Target relationships without artifacts associated with the key response actions and behavioral data (reaction time and accuracy) from the Target-Catch judgment. To further confirm that the primes were not consciously perceived, the participants who reported noticing the L1 primes would be excluded after the main test phase (three participants were excluded because of self-reported recognizing the masked primes.).

Prior to testing, there was a brief training session to ensure that the participants understood the task. The 240 trials were randomly divided into four blocks. Self-paced breaks occurred within the interval of 60 trials. The entire experiment lasted for about 40 min.

### EEG recording and ERP analyses

During the task, EEG data were collected from 64 Ag/AgCl active electrodes (10–20 system) with an actiCHamp amplifier (Brain Products GmbH, Germany). The sampling rate was 1000 Hz, and the on-line frequency range was 0.01–70 Hz. The reference and ground electrodes were Cz and Fpz, respectively. The vertical electrooculogram (VEOG) was monitored by an electrode pasted 1 cm above the left eye. All electrode impedances were maintained below 5 KΩ.

Off-line analyses were performed with Brain Vision Analyzer 2.1 software (Brain Product, Germany). All EEG data were re-referenced to the average activity of two mastoids and then filtered with an IIR bandpass filter of 0.5–30 Hz (24 dB/octave slope). The ocular artifacts were mathematically corrected by using independent component analysis (ICA) method. Based on trials with a correct response in the delayed matching-to-sample judgment, the data were segmented from − 150 to 800 ms after the target onset. Baseline correction was performed from − 150 ms to − 50 ms before the target stimulus onset. Segments with a maximally allowed amplitude of ± 75 µV were included (0.8% of the segments were excluded). The number of trials per condition after artifact rejection was 52.8 (SD = 4.9), 53.2 (SD = 4.1), 53.9 (SD = 4.0), and 53.1 (SD = 5.2), respectively. The remaining segments were averaged separately for each participant and Prime-Target condition.

According to the scalp voltage maps of the ERP difference waves (see Fig. 2), the averaged amplitude values across five representative electrode clusters, namely, the left frontal (LF: F3, F5, F7, FC3, and FC5), right frontal (RF: F4, F6, F8, FC4, and FC6), middle central (MC: FCz, C1, Cz, C2, and CPz), left parietal (LP: P3, P5, P7, PO3, and PO7), and right parietal (RP: P4, P6, P8, PO4, and PO8) electrode clusters, were calculated separately. For the averaged amplitude within each 100-ms time window from 0 to 600 ms, a repeated-measures ANOVA with within-subject factors of Prime-Target Condition (TE vs. OR vs. PR vs. UC) and electrode cluster (LF vs. RF vs. MC vs. LP vs. RP) was performed. Greenhouse–Geisser correction^51^ was applied for all within-subject measures. Partial eta squared ($\upeta_{{\text{p}}}^{2}$) is reported as a measure of effect size. Since the UC condition was used as a baseline to evaluate the cross-language lexical-semantic effects, planned pairwise comparisons concentrated on the comparisons between through-translation related priming against unrelated control with Bonferroni correction.

## Supplementary Information

Supplementary Information.

## Data Availability

The data and materials in the current study are available from the corresponding author if request.
