# Supplementary material for: Electrophysiological correlates of masked orthographic and phonological priming in Chinese–English bilinguals
Source: Sci Rep. 2022 Oct 5;12:16648. doi: 10.1038/s41598-022-21072-z (PMC9535002; doi:10.1038/s41598-022-21072-z)
Supplement: Supplementary file 1 — Supplementary Information. [file 41598_2022_21072_MOESM1_ESM.docx]

**Appendix:** The stimuli used in the experiment.

| **TE match** | | |  | **TE mismatch** | | |
| --- | --- | --- | --- | --- | --- | --- |
| **L1** | **L2** | **catch** |  | **L1** | **L2** | **catch** |
| 镜 | mirror | MIRROR |  | 包 | bag | BAD |
| 光 | light | LIGHT |  | 金 | gold | GOOD |
| 梦 | dream | DREAM |  | 床 | bed | BUD |
| 耳 | ear | EAR |  | 肉 | meat | MEET |
| 窗 | window | WINDOW |  | 画 | paint | POINT |
| 美 | beauty | BEAUTY |  | 表 | watch | WITCH |
| 水 | water | WATER |  | 头 | head | HEED |
| 碳 | carbon | CARBON |  | 爱 | love | LIVE |
| 刀 | knife | KNIFE |  | 帽 | hat | HIT |
| 哭 | cry | CRY |  | 马 | horse | HOUSE |
| 吞 | swallow | SWALLOW |  | 癌 | cancer | CANCEL |
| 砖 | brick | BRICK |  | 鹰 | eagle | EAGER |
| 腰 | waist | WAIST |  | 怒 | anger | ANGEL |
| 掌 | palm | PALM |  | 粉 | powder | PONDER |
| 花 | flower | FLOWER |  | 沟 | gap | GAW |
| 龙 | dragon | DRAGON |  | 奶 | milk | MILE |
| 酱 | sauce | SAUCE |  | 狮 | lion | LOAN |
| 舌 | tongue | TONGUE |  | 虫 | worm | WARM |
| 国 | country | COUNTRY |  | 丝 | silk | SLIM |
| 层 | layer | LAYER |  | 棋 | chess | CHASE |
| 鞋 | shoes | SHOES |  | 嘴 | mouth | MOUNT |
| 靴 | boot | BOOT |  | 锁 | lock | LOOK |
| 轮 | wheel | WHEEL |  | 银 | silver | SLIVER |
| 碟 | disk | DISK |  | 切 | cut | CAT |
| 铁 | iron | IRON |  | 泵 | pump | PUMA |
| 盘 | dish | DISH |  | 潜 | dive | DOVE |
| 臂 | arm | ARM |  | 糖 | sugar | SUPER |
| 热 | heat | HEAT |  | 疤 | scar | SCAN |
| 走 | walk | WALK |  | 篮 | basket | BASSET |
| 云 | cloud | CLOUD |  | 鼻 | nose | NOTE |

| **OR match** | | |  | **OR mismatch** | | |
| --- | --- | --- | --- | --- | --- | --- |
| **L1** | **L2** | **catch** |  | **L1** | **L2** | **catch** |
| 线 | money(钱) | MONEY |  | 怯 | law(法) | LOW |
| 骑 | chair(椅) | CHAIR |  | 坏 | cup(杯) | CAP |
| 特 | poem(诗) | POEM |  | 踝 | class(课) | GLASS |
| 相 | tear(泪) | TEAR |  | 他 | pool(池) | POOR |
| 愉 | steal(偷) | STEAL |  | 秒 | sand(沙) | SANE |
| 格 | road(路) | ROAD |  | 板 | rice(饭) | RIDE |
| 般 | boat(船) | BOAT |  | 洒 | wine(酒) | WIDE |
| 悦 | tax(税) | TAX |  | 续 | read(读) | REED |
| 低 | paper(纸) | PAPER |  | 捡 | face(脸) | FADE |
| 物 | kiss(吻) | KISS |  | 柯 | river(河) | RIVAL |
| 汁 | needle(针) | NEEDLE |  | 冷 | bell(铃) | BILL |
| 鲜 | whale(鲸) | WHALE |  | 胸 | brain(脑) | BRAND |
| 跟 | wolf(狼) | WOLF |  | 呢 | mud(泥) | MAD |
| 责 | debt(债) | DEBT |  | 能 | bear(熊) | BEER |
| 鸟 | island(岛) | ISLAND |  | 谋 | coal(媒) | COOL |
| 描 | anchor(锚) | ANCHOR |  | 搭 | tower(塔) | TOWEL |
| 借 | mistake(错) | MISTAKE |  | 准 | push(推) | PUSS |
| 揭 | drink(喝) | DRINK |  | 粗 | rent(租) | REND |
| 约 | medicine(药) | MEDICINE |  | 施 | drag(拖) | DROP |
| 根 | hate(恨) | HATE |  | 梳 | flow(流) | FLAW |
| 贾 | ticket(票) | TICKET |  | 梅 | sea(海) | SEE |
| 陈 | frozen(冻) | FROZEN |  | 霜 | snow(雪) | SNOB |
| 汉 | power(权) | POWER |  | 创 | gun(枪) | GIN |
| 爷 | axe(斧) | AXE |  | 宠 | cage(笼) | CAFE |
| 姻 | smoke(烟) | SMOKE |  | 场 | soup(汤) | SOUR |
| 狙 | group(组) | GROUP |  | 级 | suck(吸) | SICK |
| 村 | tree(树) | TREE |  | 汗 | liver(肝) | LIVEN |
| 猪 | gamble(赌) | GAMBLE |  | 狠 | wave(浪) | WAKE |
| 驼 | snake(蛇) | SNAKE |  | 挑 | peach(桃) | PEACE |
| 控 | dig(挖) | DIG |  | 监 | salt(盐) | SALE |

| **PR match** | | |  | **PR mismatch** | | |
| --- | --- | --- | --- | --- | --- | --- |
| **L1** | **L2** | **catch** |  | **L1** | **L2** | **catch** |
| 深 | sound(声) | SOUND |  | 查 | tea(茶) | TEE |
| 脚 | angle(角) | ANGLE |  | 谷 | bone(骨) | BOND |
| 晚 | bowl(碗) | BOWL |  | 壶 | lake(湖) | LATE |
| 瞎 | shrimp(虾) | SHRIMP |  | 乘 | town(城) | DOWN |
| 菊 | orange(橘) | ORANGE |  | 压 | duck(鸭) | DUKE |
| 数 | mouse(鼠) | MOUSE |  | 加 | home(家) | HOPE |
| 基 | chicken(鸡) | CHICKEN |  | 辞 | word(词) | WORK |
| 恶 | hunger(饿) | HUNGER |  | 页 | leaf(叶) | LEAD |
| 首 | hand(手) | HAND |  | 东 | winter(冬) | WINNER |
| 徒 | picture(图) | PICTURE |  | 栋 | hole(洞) | HOLD |
| 典 | dot(点) | DOT |  | 忠 | clock(钟) | CLICK |
| 骄 | teach(教) | TEACH |  | 输 | book(书) | BOOM |
| 西 | knee(膝) | KNEE |  | 语 | rain(雨) | RAIL |
| 难 | south(南) | SOUTH |  | 阳 | sheep(羊) | SHEET |
| 瞧 | bridge(桥) | BRIDGE |  | 原 | circle(圆) | CIRCUS |
| 常 | length(长) | LENGTH |  | 核 | box(盒) | BOW |
| 做 | seat(座) | SEAT |  | 封 | wind(风) | WINK |
| 平 | bottle(瓶) | BOTTLE |  | 欺 | wife(妻) | WISE |
| 亡 | king(王) | KING |  | 讲 | prize(奖) | PRIDE |
| 曲 | marry(娶) | MARRY |  | 时 | stone(石) | STORE |
| 碑 | sad(悲) | SAD |  | 感 | dare(敢) | DARN |
| 割 | song(歌) | SONG |  | 官 | close(关) | CLOVE |
| 游 | oil(油) | OIL |  | 闭 | coin(币) | COIL |
| 兵 | ice(冰) | ICE |  | 囚 | ball(球) | BALD |
| 剑 | arrow(剑) | ARROW |  | 捉 | desk(桌) | DECK |
| 齿 | ruler(尺) | RULER |  | 目 | wood(木) | WOOL |
| 瘦 | beast(兽) | BEAST |  | 糕 | height(高) | WEIGHT |
| 贝 | back(背) | BACK |  | 卖 | wheat(麦) | WHEAL |
| 余 | fish(鱼) | FISH |  | 陆 | deer(鹿) | DEAR |
| 步 | cloth(布) | CLOTH |  | 弹 | egg(蛋) | EGO |

| **UC match** | | |  | **UC mismatch** | | |
| --- | --- | --- | --- | --- | --- | --- |
| **L1** | **L2** | **catch** |  | **L1** | **L2** | **catch** |
| 雷 | video(视频) | VIDEO |  | 泉 | type(类) | TYRE |
| 文 | coach(教练) | COACH |  | 网 | train(火车) | TRAIL |
| 伞 | candy(糖) | CANDY |  | 蜡 | shape(形) | SHARP |
| 锅 | tooth(牙) | TOOTH |  | 车 | danger(危险) | DANCER |
| 看 | rocket(火箭) | ROCKET |  | 店 | apple(苹果) | APPLY |
| 食 | pencil(笔) | PENCIL |  | 菜 | hire(雇) | HIDE |
| 军 | failure(失败) | FAILURE |  | 笔 | land(陆地) | LANE |
| 修 | honour(光荣) | HONOUR |  | 唱 | earth(地球) | EARLY |
| 园 | ocean(海) | OCEAN |  | 迷 | dust(尘) | DUTY |
| 棉 | issue(问题) | ISSUE |  | 果 | weapon(武器) | WEAKEN |
| 狗 | bomb(炸) | BOMB |  | 天 | season(季) | REASON |
| 音 | theory(理论) | THEORY |  | 扇 | faith(信念) | FAINT |
| 邮 | moon(月) | MOON |  | 屋 | foot(足) | FOOL |
| 面 | lawyer(律师) | LAWYER |  | 壳 | market(市场) | MARKER |
| 电 | wall(墙) | WALL |  | 夏 | court(法庭) | COUNT |
| 鸽 | farmer(农民) | FARMER |  | 笑 | drug(药) | DRUM |
| 师 | cheese(奶酪) | CHEESE |  | 区 | butter(黄油) | BUTLER |
| 类 | gift(礼) | GIFT |  | 豆 | card(卡) | CARE |
| 灰 | leg(腿) | LEG |  | 烦 | wallet(钱包) | WALKER |
| 买 | coat(外套) | COAT |  | 坟 | food(食物) | Foil |
| 抖 | boss(老板) | BOSS |  | 静 | flag(旗) | FLAP |
| 庙 | cook(煮) | COOK |  | 笛 | guard(守卫) | GOURD |
| 观 | poison(毒) | POISON |  | 景 | hair(头发) | HEIR |
| 答 | joke(玩笑) | JOKE |  | 鬼 | blood(血) | BLOOM |
| 琴 | driver(司机) | DRIVER |  | 猫 | luck(运气) | LICK |
| 梨 | soul(灵魂) | SOUL |  | 玩 | copy(抄袭) | COPE |
| 漂 | code(代码) | CODE |  | 灯 | beef(牛肉) | BEEP |
| 眼 | air(空气) | AIR |  | 楼 | park(停) | BARK |
| 柜 | monkey(猴) | MONKEY |  | 江 | bean(豆) | BEAT |
| 跳 | smile(笑) | SMILE |  | 衣 | mark(记号) | MAKE |

Note: TE=translation equivalents, OR=orthographically related through translation, PR=phonologically related through translation, UC=unrelated control.
